# Blood Supply and Draining Vein of Cerebellectomy Area

**DOI:** 10.21315/mjms-01-2026-072

**Published:** 2026-02-28

**Authors:** Lee Wei Lun, Zamzuri Idris, Muhammad Ihfaz Ismail

**Affiliations:** 1 Department of Neurosciences, School of Medical Sciences, Universiti Sains Malaysia, Health Campus, Kelantan, Malaysia; 2 Brain and Behaviour Cluster, School of Medical Sciences, Universiti Sains Malaysia, Health Campus, Kelantan, Malaysia; 3 Department of Neurosciences and Brain Behaviour Cluster, Universiti Sains Malaysia Specialist Hospital, Universiti Sains Malaysia, Health Campus, Kelantan, Malaysia

Dear Editor,

We read with interest the letters to the editor titled “Brain Shift Patterns: Upward, Lateral and Downward Herniation, Its Correlation with Clinical Patterns in Acute Intracranial Pathologies and Management” (https://ejournal.usm.my/mjms/article/view/mjms_vol32-no6-2025_16) by Nur Nazleen Said Mogutham, Jafri Malin Abdullah, Suzuanhafizan Omar, Mohammad Iskandar Sa’uadi, and Sharifah Nawal Syed Jaafar published in the *Malaysian Journal Medical Sciences* Volume 32(6), 2025 where downward and upward herniation were discussed with relevant neuroanatomy (1). The neurosurgical dissection for a cerebellectomy was also demonstrated. We would like to elaborate on the relevant arteries and veins ligated/clipped/coagulated/sacrificed during this unilateral cerebellectomy procedure to complement the author’s operative discussion.

From an arterial perspective, the posterior inferior cerebellar arteries (PICAs) and superior cerebellar arteries (SCAs), which constitute the two largest cerebellar arterial pairs, demonstrate a functionally important division into medial and lateral territories. The medial branches predominantly supply the vermis and paravermian regions, with extensions to the deep cerebellar nuclei and cerebellar peduncles, and therefore should be preserved (2, 3). In contrast, the lateral or cortical branches mainly supply the cerebellar hemispheres and may be sacrificed during cerebellar hemispherectomy. The anterior inferior cerebellar arteries (AICAs), by comparison, supply a relatively limited territory involving the anterior inferior cerebellum and flocculus. They do not divide into major medial and lateral cerebellar branches but instead give off variable small twigs to adjacent structures (2–4).

More specifically, within the PICA territory, the telovelotonsillar (p4) segment supplies the vermis, inferior cerebellar peduncle, choroid plexus, and inferior medullary velum, and should be preserved (3–5). The cortical (p5) segment (Figure 1) gives rise to medial and lateral cortical branches. The medial PICA branches, which include the median and paramedian vermian arteries, are critical for vermian perfusion and should be preserved. The lateral PICA branches— namely the tonsillar and medial, intermediate, and lateral hemispheric arteries—supply the inferior two-thirds of the biventeral lobule, most of the inferior semilunar lobule, and the anterolateral portion of the tonsil, and may be sacrificed during hemispherectomy (2, 3, 6). Importantly, cortical branches arising near the superior pole of the tonsil may give rise to vessels supplying the dentate nucleus; these branches should be identified and preserved during dissection of the lateral PICA branches whenever possible.

With respect to the SCA, the cerebellomesencephalic (s3) segment supplies the superior cerebellar peduncles and must be preserved. The SCA typically divides early into rostral and caudal branches (2, 3). The rostral SCAs predominantly supply the superior vermis, dentate nucleus, and other deep cerebellar nuclei and should be preserved. The caudal SCAs mainly supply the lateral cerebellar hemispheres, including the anterior lobe, simplex lobule, and superior portion of the semilunar lobule, and may be sacrificed during cerebellectomy (2, 3, 7). When dissecting near the paravermian region, cortical arteries supplying paravermian surfaces lateral to the vermis(mostly from rostral SCAs) may give rise to precerebellar branches to the dentate and deep nuclei; these are important deeper perforators and need to be preserved to avoid neurological dysfunction (3, 6).

However, contemporary anatomic and radiologic analyses have refined this classical understanding. Venier and Lanzino (4) described that the dentate nucleus received its supply predominantly by direct perforating branches arising from the hemispheric (caudal/lateral) trunk of the superior cerebellar artery, rather than the rostral trunk as explained by Rhoton (3, 4). These perforators course through the cerebellar white matter and may not be apparent on the cerebellar surface, placing them at risk during hemispheric debulking or deep white matter dissection. Inadvertent sacrifice during cerebellectomy may lead to dentate ischaemia and cerebellar outflow dysfunction. This highlights the need for caution not only when preserving rostral SCA branches near the vermis, but also when handling caudal SCA trunks during cerebellar hemispherectomy (Figure 2).

For the AICA, the flocculopeduncular (a3) segment supplies the flocculus and middle cerebellar peduncle and should be preserved (Figure 3). The cortical (a4) segment demonstrates marked variability, ranging from a small contribution to the flocculus and adjacent petrosal surface to supplying the entire petrosal surface of the cerebellum (2–5). Cortical surface branches running within the superior and inferior lip of the cerebellopontine fissure may be sacrificed when required for hemispheric resection.

Venous considerations are equally important. Superficial cerebellar veins are arranged according to the tentorial, suboccipital, and petrosal groups (2, 3). Inferior and superior hemispheric of the tentorial and suboccipital groups may be sacrificed during hemispherectomy, whereas inferior and superior vermian veins should be preserved. Tributaries from tonsillar veins draining into the vermian system, however, can usually be sacrificed safely (3, 7). Deep cerebellar veins should be preserved, with the notable exception of the precentral cerebellar vein (vein of the cerebellomesencephalic fissure), where its connection to the galenic venous system can be interrupted to improve surgical exposure, provided its collaterals are intact (Figure 4). Regarding bridging veins, sacrifices are frequently required and are generally considered safe (7). Superior cerebellar bridging veins, which usually arise from hemispheric veins of the tentorial and suboccipital surfaces and drain into the torcular or tentorial venous sinuses, may also be sacrificed. Occasionally, bridging veins arise from the inferior vermis or cerebellar hemisphere and drain into the occipital sinuses; these veins can generally be sacrificed without adverse clinical effects (3, 7, 8).

We hope that these additional anatomical considerations regarding cerebellar arterial and venous management will further enhance the clarity and practical utility of surgical approaches to cerebellar hemispherectomy.[Fig f5-14mjms3301_le]

## Figures and Tables

**Figure 1:**
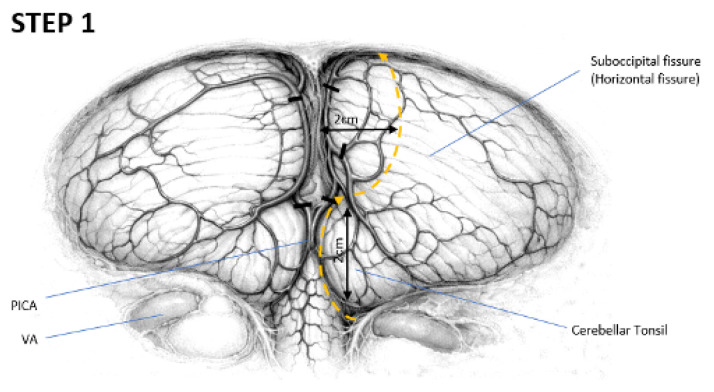
Illustration of the suboccipital surface with the PICA supply The cortical (p5) segment is ligated and sectioned during cerebellectomy. Be aware of any cortical branches arising near the superior pole of the tonsil which may supply the inferior dentate nucleus. Black stripes are the suggested location for arterial interruption or clip placement during cerebellectomy. Yellow dashed arrows show the direction of cerebellectomy.

**Figure 2:**
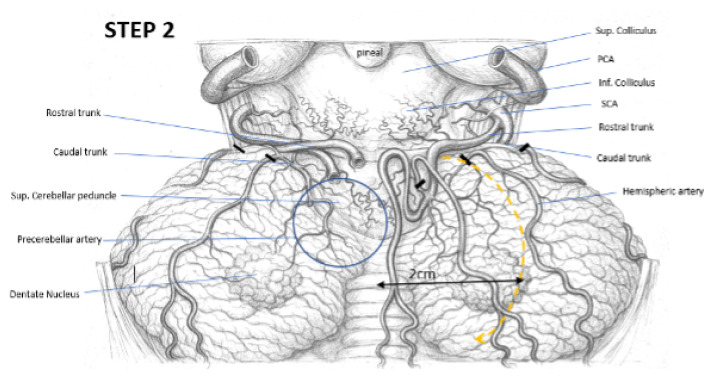
Illustration of the tentorial surface with the SCA The blue star highlights the small perforating precerebellar branches arising from the SCA that supply the deep nuclei and should be preserved. Black stripes are the suggested location for arterial interruption or clip placement during cerebellectomy. Yellow dashed arrows show the direction of cerebellectomy.

**Figure 3:**
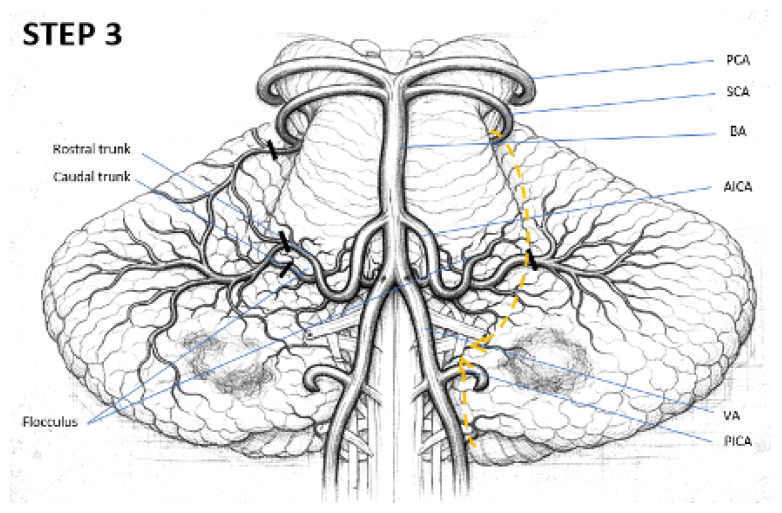
Illustration of the petrosal surface with the AICA supply The flocculopeduncular (a3) segment is preserved and the cortical (a4) segment can be clipped or ligated during cerebellectomy. Black stripes are the suggested location for arterial interruption or clip placement during cerebellectomy. Yellow dashed arrows show the direction of cerebellectomy.

**Figure 4:**
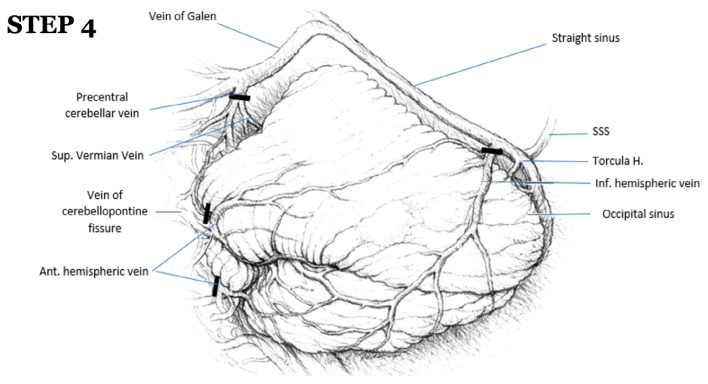
Illustration of the lateral surface of the cerebellar with the three draining groups of veins Hemispheric and bridging veins can be interrupted during cerebellectomy. Black stripes are the suggested location for its venous interruption during cerebellectomy.

**Figure 5 f5-14mjms3301_le:**
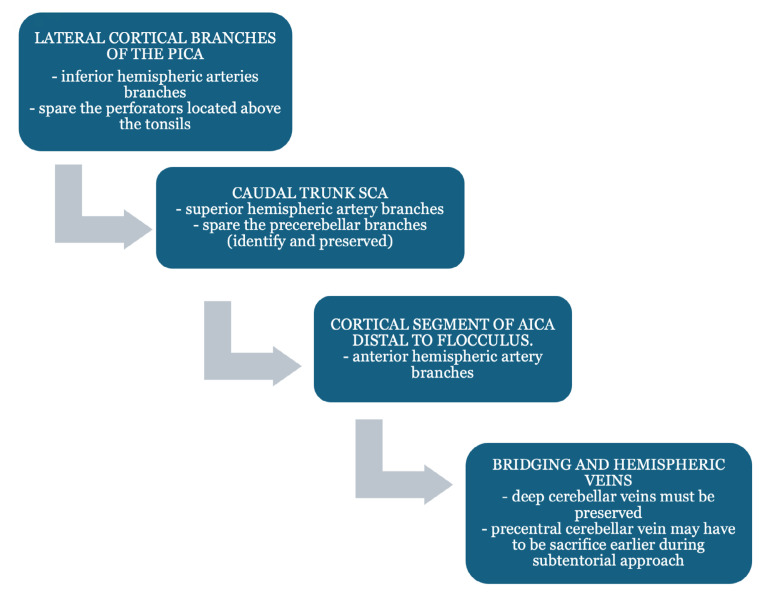
Flow chart of the order of vessels ligation/section during the process of cerebellectomy.

## References

[1] Said Mogutham NN, Abdullah JM, Omar S, Sa’uadi MI, Syed Jaafar SN (2025). Brain shift patterns: upward, lateral and downward herniation, its correlation with clinical patterns in acute intracranial pathologies and management. Malays J Med Sci.

[2] Manto MU, Gruol DL, Schmahmann JD, Koibuchi N, Sillitoe RV (2020). Handbook of the cerebellum and cerebellar disorders.

[3] Rhoton AL (2019). Rhoton’s cranial anatomy and surgical approaches.

[4] Venier A, Lanzino G, Robert T, Bonasia S, Bojanowski MW (2015). Embryology and variations of superior cerebellar artery. Anatomy of cranial arteries: embryology and variants.

[5] Matsushima T (2015). Microsurgical anatomy and surgery of the posterior cranial fossa: surgical approaches and procedures based on anatomical study.

[6] Ramos A, Chaddad-Neto F, Dória-Netto HL, Campos-Filho JM, Oliveira E (2012). Cerebellar anatomy as applied to cerebellar microsurgical resections. Arq Neuropsiquiatr.

[7] Elhammady MS, Heros RC (2013). Cerebral veins: to sacrifice or not to sacrifice, that is the question. World Neurosurg.

